# Challenges and Opportunities for Universal Health Coverage in South Asia: A Scoping Review

**DOI:** 10.1177/10105395241296653

**Published:** 2024-11-15

**Authors:** Jayendra Sharma, Milena Pavlova, Wim Groot

**Affiliations:** 1 Department of Health Services Research, CAPHRI, Maastricht University Medical Center, Faculty of Health, Medicine and Life Sciences, Maastricht University, Maastricht, Netherlands

**Keywords:** South Asia, universal health coverage, universal coverage, service delivery, financial protection

## Abstract

With a significant proportion of the population facing considerable deficits in access to health care and financial protection, progress toward universal health coverage (UHC) continues to be a challenge in South Asia. The objective of this scoping review is to examine the challenges and opportunities for UHC in South Asia. We used the six-stage Arksey and O’Malley methodological framework for investigation and the Preferred Reporting Items for Systematic Reviews and Meta-analysis extension for Scoping Reviews checklist to structure and report the review. A systematic search retrieved 2776 records from three databases and 13 gray literature sources, of which 27 records were reviewed. Frequently emerging challenges include underfunding of the health system, the nascent stage or inadequate coverage of the social protection or insurance system, fragmentation in the health system, the inability to effectively regulate the private sector, a health system that is unprepared to effectively address non-communicable diseases, and concerns about the quality of and equality of access to health care. While a diversity of challenges, mostly driven by the country-specific context, continue to falter progress toward UHC in the South Asia region, several consistent themes emerge. Considering this as an initial attempt to map the existing literature, we recommend future research to examine how the challenges and priorities evolve over time.

## What We Already Know

- With deficits in access to health care and financial protection, progress toward UHC continues to be a challenge in South Asia.
- Among all regions, South Asia had the second lowest level of UHC service coverage and the highest level of catastrophic health spending in 2021.
- The COVID-19 pandemic has exacerbated existing weaknesses in health systems in the region.

## What This Article Adds

- This scoping review maps the existing literature on UHC challenges and opportunities in South Asia.
- A diversity of challenges continues to falter progress toward UHC in South Asia.
- While challenges and opportunities are country-specific, there are several common themes.

## Introduction

With the United Nations political declaration on universal health coverage (UHC) in 2019^1^ and its adoption by several multilateral global health agencies, UHC has clearly gained momentum and global commitment. UHC is generally understood as a situation where everyone, irrespective of their ability to pay, receives the health services they need in a timely fashion, in accordance with quality standards and without suffering any undue financial hardship as a result of receiving the care.^2,3^

Following the adoption of the UHC goals at the global and national levels, measurement and monitoring frameworks have evolved and matured both in terms of single-country investigations and globally standardized measurement. In 2014, the World Health Organization (WHO) and the World Bank released the UHC monitoring framework,^3,4^ which proposed methodology for tracking country and global progress toward UHC. Notwithstanding the progress in monitoring UHC, given the wide scope of this concept and its complex interplay with political, legal, economic, social, and commercial factors, it is important to document the contextual ecosystem of associated challenges and issues. Among some noteworthy recent initiatives, the comprehensive health systems performance assessment framework for UHC^5^ and the health financing progress matrix^6^ are useful tools to enable countries to understand key strengths and shortcomings. This paper outlines an approach to mapping evidence on progress toward UHC based on the WHO definition and guidance on UHC progress,^2-4^ and applies it to South Asia.

The South Asia region, defined by membership of the South Asian Association for Regional Cooperation, comprises eight countries—Afghanistan, Bangladesh, Bhutan, India, Maldives, Nepal, Pakistan, and Sri Lanka. The region is home to about a quarter of the world’s population. Together with low levels of coverage of essential health services and financial risk protection, inequality in access to health care remains a serious issue for most countries in South Asia.^7,8^ In the latest global monitoring report, the South Asia region had the second lowest level of UHC service coverage, following Sub-Saharan Africa, and the highest level of catastrophic health spending among all regions around the globe.^9^ With a significant population pool of more than a quarter of the world population, and considerable deficits in health access and financial protection, South Asia assumes a critical space in the global health stage. Without significant progress in these countries, global progress on UHC and Sustainable Development Goals would be severely challenged.

While sharing elements of common history, geography, and heritage, South Asia is an extremely diverse region, with large variations in demographics and economic and health indicators (see Supplemental Material, p 2 for comparison among these countries). South Asian countries enhanced the UHC service coverage index from 28 in 2000 to 59 in 2021.^9^ Sri Lanka, Maldives, and Bhutan were the highest performers, scoring over 50, while India, Nepal, Pakistan, and Afghanistan performed lower (Supplemental Material, p 2). In terms of catastrophic payments for health care, the proportion of the population spending more than 10% of their household income on health care stood at 17.7% against the global average of 13.5%.^9^ Afghanistan, Bangladesh, and India recorded the highest level of catastrophic incidence (Supplemental Material, p 2). Socio-economic inequities in access to essential health care services and health care quality are other prominent challenges in South Asia.^10^ The COVID-19 pandemic has exacerbated existing weaknesses in health systems in the region, highlighting a critical lack of health care professionals, infrastructure, and commodities, leading to significant disruption of essential services.^10,11^ There are calls for future pandemic preparedness and response mechanisms to simultaneously leverage global health security and UHC to ensure long-term resilience and equity.^12^

Previous regional studies have focused on quantitative assessment of UHC indicators^7,13^ or have been confined to specific population groups.^8^ There is a need for a more comprehensive understanding of the country-specific and cross-country challenges in South Asia. In view of the dynamic nature and progressive realization of UHC, the priorities are expected to differ among countries. The objective of this scoping review was, therefore, to examine the challenges and opportunities for UHC in South Asia by mapping the available literature on UHC in this region and identifying literature gaps. In contrast to earlier studies, we extracted key themes from the globally accepted WHO definition and guidance on UHC progress and applied them to map and compare evidence across countries. We believe that this can be an important perspective in the discussion on how to standardize future assessments and comparative analysis of UHC.

## Methods

### Review Approach

Considering the multi-dimensionality of the concept of UHC and the heterogeneity in the literature and evidence sources, we adopted the rapidly evolving methodological approach of scoping reviews. Scoping reviews systematically map the literature available on a particular topic and identify key concepts, theories, sources of evidence, and gaps in research.^14^ We used the six-stage methodological framework of Arksey and O’Malley^15^ and considered the framework enhancements and recommendations provided by several subsequent projects and studies.^16-20^ We used the Preferred Reporting Items for Systematic Reviews and Meta-analysis extension for Scoping Reviews (PRISMA-SCR) checklist^21^ to structure and report the review results. The review protocol used in the study is provided in the Supplemental Material (p 15-17).

### Identifying the Main Concepts in the Research Question

We adopted the Population, Concept, and Context framework,^17^ to identify the main concepts in the research question, clarify inclusion criteria, and inform the search strategy for the review (Table 1). We adopted the widely used definition of UHC,^2^ which emphasizes that all people have access to the full range of quality health services they need, when and where they need them, without financial hardship.

**Table 1.** PCC Framework.

Population (P)	All residents of all ages of countries in South Asia
Concept (C)	Definition of universal health coverage and guidance by the World Health Organization
Context (C)	National level: single country, multiple countries, or collectively, the South Asia region

### Identifying Relevant Studies

#### Inclusion and exclusion criteria

Along with peer-reviewed journal articles, studies and reports that were globally standardized by the WHO or policy documents from national governments were included. Articles and reports needed to address the overall UHC progress and challenges or at least one of the identified thematic components discussed below. All study designs were considered. We excluded articles that focused on a specific aspect of UHC, examined a single or a set of specific disease or health system issues, focused on subnational levels, and those using non-English language and published prior to 2010.

#### Search strategy

The identification of the literature was conducted through three separate approaches: searches in electronic databases, examining webpages of ministries of health and multilateral institutions for country-specific publications, as well as iterative reviews of reference lists of papers. The database search strategy, peer-reviewed by a librarian, was implemented on three electronic databases (PubMed, Web of Science, and Scopus) on June 13, 2023, and an updated search was performed on December 13, 2023. In addition, manual searches were conducted for Health in Transition reports of the WHO and reference list for those countries where relevant contents were inadequate (Afghanistan and Maldives). The complete search strategy is illustrated in the Supplemental Material (p 6).

### Study Selection

Using EndNote 20, the selection of studies was made in three screening stages. Stage 1 involved the removal of duplicates, which entailed both software-based and manual deduplication of records. Stage 2 screening involved reading titles and abstracts of all publications that passed stage 1 to assess their relevance to the topic based on the inclusion criteria. In stage 3, the full texts of the publications that passed stage 2 screening were assessed against the inclusion criteria for the final selection of studies to be included in the review. Screenings at stages 2 and 3 were done independently by two reviewers. Differences in opinion were discussed and resolved prior to moving to the following stage. The final screening stage included the review of the reference lists of publications that passed stage 3.

### Charting the Data

Following the recommendations for the extraction, analysis, and presentation of results in scoping reviews,^22^ a standardized data extraction tool was developed in Microsoft Excel and pilot-tested to chart the results of the review. We adopted the generally accepted WHO definition of UHC progress (the UHC cube) and related WHO guidance^2^ as well as WHO global monitoring framework^3,4^ in identifying six broad themes to describe and classify the possible array of challenges for countries in their progress toward UHC. Specifically, we identified three themes based on the prerequisites for achieving UHC defined by WHO: (1) policy and governance beyond the health sector; (2) policy and governance within the health sector; (3) monitoring progress toward UHC; and three themes based on the cube dimensions proposed by WHO to define the progress toward UHC: (4) effective coverage of services; (5) financial protection; (6) access for all people in need. The extracted data were discussed iteratively among authors to resolve discrepancies and ensure the consistency and robustness of the analysis.

### Collating, Summarizing, and Reporting the Results

We profiled the selected articles summarizing the origin, time, objectives, and design of the studies or reports along with simple descriptive analysis of the literature. We then delved into basic qualitative content analysis of the findings along the six pre-identified categories through a deductive analysis and extraction approach.^22^ We did not assess the research quality of the publications since we included non–peer-reviewed publications as well. The results were presented narratively and illustrated with tables. Preferred Reporting Items for Systematic Reviews and Meta-analysis extension for Scoping Reviews checklist was used to ensure adequate reporting on the review (Supplemental Material, p 3).

## Results

### Selection and Profile of the Literature

**Figure 1.**
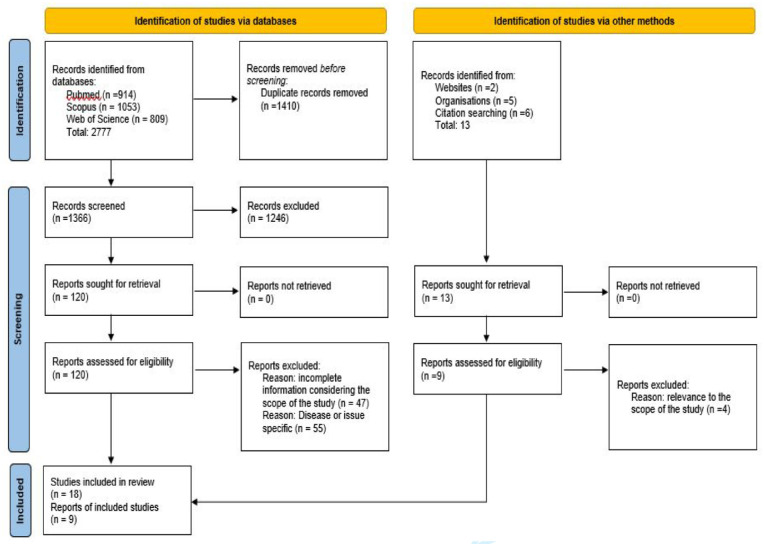
PRISMA 2020 flow diagram for new systematic reviews which included searches of databases, registers, and other sources.

The search process retrieved 2777 records from the three databases, including 80 additional articles retrieved from an updated search. From this pool, 1366 articles were selected for screening after duplicates removal. A total of 1246 articles were excluded during the title and abstract screening process, and an additional 102 articles were excluded after full-text review. This resulted in 18 articles selected from the database search. The manual search through webpages of government and international organizations, Google search, and snowballing through the reference list yielded an additional nine articles and reports. A total of 27 articles and reports were included in the scoping review. The result of the search, screening, and selection process is illustrated in Figure 1 as per the PRISMA 2020 flow diagram.^23^

### General Characteristics of the Publication Included in the Review

Table 2 outlines the profile of the publications included in the review. A large majority of the studies selected were articles addressing individual countries (*n* = 23), while some articles (*n* = 4) addressed multiple countries. Bangladesh and India led the list of countries with publications reviewed. The periods 2018-2019 and 2022-2023 were the most frequent publication periods for the selected studies. The number of publications has gradually increased over the years, with a significant drop during 2020-2021 coinciding with the COVID-19 pandemic. While most of the articles were reviews (*n* = 12) and perspective or commentary (*n* = 7), the eligible articles also included four quantitative investigations, two mixed methods studies, one qualitative investigation, and one government report.

**Table 2.** Profile of the Publications Included in the Analysis (*N* = 27).

Characteristics	Categories	*N* (%)	References
Country coverage	Single country	23 (85)	^24-46^
	Multiple countries	4 (15)	^7,8,13^
Year	2014-2015	3 (11)	^29,33,47^
	2016-2017	5 (19)	^7,8,30,38,45^
	2018-2019	9 (33)	^13,25,27,28,34,36,42,43,44^
	2020-2021	3 (11)	^37,39,46^
	2022-2023	7 (26)	^24,26,31,32,35,40,41^
Countries	Afghanistan	3 (11)	^24,25,34^
	Bangladesh	4 (15)	^27,33,43,44^
	Bhutan	2 (7)	^29,30^
	India	4 (15)	^32,35,40,45^
	Maldives	2 (7)	^31,39^
	Nepal	2 (7)	^28,36^
	Pakistan	3 (11)	^26,41,46^
	Sri Lanka	3 (11)	^37,38,42^
	Multi-country	4 (15)	^7,8,13,47^
Type of publication	Review	8 (30)	^8,32,28,36,39,42,44,47^
	HiT reports (review)	4 (15)	^30,33,35,37^
	Quantitative	4 (15)	^7,13,43,45^
	Qualitative	1 (4)	^40^
	Mixed methods	2 (7)	^27,29^
	Perspective/comment	7 (26)	^24,26,31,34,38,41,46^
	Government report	1 (4)	^25^

### Challenges and Opportunities for UHC

#### Policy and governance beyond the health sector

Underfunding of the health care sector and investments in health that do not match the increase in demand or the economic growth emerged as the predominant challenge for UHC reported in the studies with 67% of the articles reviewed reporting this as a challenge. Other country-specific challenges included high poverty rates in Afghanistan, Bangladesh, India, Nepal, and Pakistan,^7^ a deteriorating humanitarian situation, volatile economic growth, national security issues marked by conflict and violence in Afghanistan,^24,25,47^ interruption of UHC initiative due to political and economic instability in Pakistan,^26^ outdated public financial management system in Bangladesh,^27^ government instability, highly politicized bureaucracy, poor governance structures in Nepal,^28^ and accelerating rates of migration, urbanization, and youth unemployment, and transition toward a market-driven society in Bhutan.^29,30^ In addition, significant threats to health due to climate change and natural disasters have been documented in Maldives,^31^ Afghanistan,^24^ and Bhutan.^30^

#### Policy and governance within the health sector

Among the common challenges to UHC were fragmentation in the health system and services^32,33,34,47^ and weak regulatory oversight of the private sector.^25,27-30,32,33,35,36^ The challenge of weak regulatory oversight of the private sector was not limited to countries which have traditionally featured mixed health systems but also those that were transitioning from a public sector–dominated health system toward more engagements of the private sector. Increases in health-seeking behavior have resulted in cost pressures on governments in Bangladesh^27^ and Bhutan.^30^ Other challenges included overwhelming dependence on development and humanitarian partners in Afghanistan,^24,34,47^ the existence of vertical programs and health system that have not evolved appropriately to meet the changing demands due to the demographic and epidemiological transitions in Sri Lanka,^37^ as well as dual affiliation of health workers in public and private sectors leading to inefficiencies and suboptimal services in the public sector in Sri Lanka.^38^ In Bhutan and Maldives, difficult geographical features, dispersed population settlements, and heavy reliance on the import of human resources and medical supplies continue to pose challenges to health sector policies and stewardship.^30,31,39^

Poor governance of the health sector in most countries in the region contributes to low-quality health care.^8^ Centralized governance structure and weak management of decentralized health systems were reported in Afghanistan,^47^ Nepal,^28^ India,^40^ and Pakistan.^41^ In addition, issues related to accountability and corruption in the health sector were documented in Afghanistan and Pakistan,^47^ as well as in Bangladesh.^27,33^

#### Effective coverage of services for all people in need

As evidenced in the reviewed publications, South Asian countries vary substantially in the coverage of essential services,^7^ with Afghanistan featuring among the worst levels of coverage of basic health interventions in the world.^34^ While UHC is being discussed by governments in the region, this has not translated into health service coverage figures, and where reported coverage of services is good, quality of care is often low.^8^ The issue of foregone health care is an issue across the region.^7^

The challenges are multifaceted, ranging from resources to policies. Among the most prominent ones documented were: shortages and maldistribution of health workers,^24,26-28,30-33,35,37,38,40,41,47^ inadequate availability of health infrastructure and supplies,^26,28,32-35,38,41,42,47^ suboptimal quality of care,^27,30,33,35,36,37^ gaps in critical health interventions, particularly related to non-communicable diseases (NCDs),^24,25,28,32,33,37-39,41,43^ a lack of balance between different levels of care,^26,30,37,40^ weak capacities of decentralized service delivery units,^28,33,40,41^ and geographical and logistical challenges.^29,30,31,34,43^ In addition, challenges and disruptions in implementing an essential package of service or ongoing UHC programs owing to the larger political, economic, or humanitarian situation in Afghanistan and Pakistan,^24,26^ continued predominance of vertical programs in Bangladesh and Sri Lanka,^33,37^ and bypassing of lower level care in Bhutan and Sri Lanka were documented.^30,37^ About 74% of the studies reported inequalities in access to health care owing to economic status, gender, or geographical and residential determinants as major challenges to UHC progress. In Sri Lanka, access to health care was poor among working-age men.^37,38^ Additional challenges with population vulnerabilities include caste, social status, ethnicity, and religion in India and Bangladesh.^27,32,40^

#### Financial protection

With six out of eight countries meeting more than half of their health care costs through household and individual out-of-pocket expenditures, financial protection is a major area of concern in the region. High levels of out-of-pocket expenditure for health care put a heavy financial burden on households, resulting in catastrophic health expenditure and impoverishment. For instance, the incidence of catastrophic payment in Bangladesh was the highest in the Asia Pacific region,^33^ and health care costs were the single biggest cause of debt in India^32^ and the foremost cause of pushing people into the poverty trap in Pakistan.^41^ Spending on medicines was the dominant component of out-of-pocket expenditure on health care, exceeding 70%, in several countries of the region.^13,33^

The predominant challenge pertained to a nascent stage or inadequate coverage of social protection or insurance.^25,27,28,33,35,36,40-43^ Some of the major country-specific challenges include fragmented social security schemes and donor aid being dispersed, inconsistent, and uncoordinated in Nepal,^28,36^ coordination issues among the center and provinces in resource allocation in Pakistan,^47^ politically driven health financing structure and heavy reliance on medical travel abroad in Maldives,^31,39^ the emergence of a third tier where out-of-pocket payments result from the use of public sector services in Sri Lanka,^38^ poorly designed public financial management or other provider payment mechanisms documented in Bangladesh and India,^27,40^ a pervasive sociocultural barrier against insurance in Bangladesh,^27^ a decline in health investments relative to the growth in the national economy and an increasing trend of indirect expenditure for health care in Bhutan,^29,30^ and the sustainability concerns on predominantly donor-financed health system in Afghanistan.^25,47^

#### UHC monitoring

We observe a paucity in the literature on comprehensive assessments of UHC at national and subnational levels. This closely confirms earlier reviews which attributed the paucity to inadequate data and tracking systems in many South Asian countries^7^ and the unavailability of recent data on financial protection for all South Asian countries.^8^ Country assessments depend predominantly on the UHC global monitoring system of the WHO and the World Bank. The availability of reliable data continues to be a key concern in politically fragile countries.^34^ Where countries have adopted UHC monitoring into their national health planning and monitoring system, there are gaps in the comprehensiveness of indicators,^38,44^ or the absence of subnational level monitoring has tended to mask deep spatial and localized inequities in health care access.^29^ Researchers within the region have also highlighted differences in opinion on the definition and goal of UHC and the fact that the estimation of a composite indicator is fraught with problems which could be normative and statistical in nature.^45^

#### Recommendations reported in the publications

The most frequent recommendations and strategies for UHC in the region included strengthening the service package and delivery system. This was followed by the need to increase investment levels, a revision of health financing policies and modalities, and the need to enhance the role of the public sector in health. Enhancing monitoring and accountability for quality of care and building the health system readiness for NCDs assume the fourth rank in the priorities for UHC in the region. Table 3 outlines these recommendations by themes and countries.

**Table 3.** Summary of Recommendations from the Reviewed Publications (*N* = 27).

	Recommendations/strategies	*n* (%)	Study countries
1	Service package and delivery redesign	15 (12.6)	Sri Lanka,^37,38^ India,^40,44^ Pakistan,^41,46^ Bhutan,^29,30^ Nepal,^36^ Bangladesh,^33,43^ Maldives,^31,39^ Multi-country^7,47^
2	Increase investments in health	14 (11.8)	Sri Lanka,^37,42^, Pakistan,^26,41^ Bhutan,^29,30^ Bangladesh,^43^ India,^35,40^ Afghanistan,^24,25^ Multi-country^7,8,47^
3	Revisit health financing modalities and policies	12 (10.1)	Nepal,^28,36^ Bangladesh,^27,33^ Afghanistan,^25,34^ India,^35,40^ Maldives,^39^ Multi-country^7,8,13^
4	Enhance the role of the public sector	11 (9.2)	Sri Lanka,^37,42^ Nepal,^28^ India,^32^ Bangladesh,^27,43^ Afghanistan,^25,34^ Pakistan,^26,46^ Multi-country^7^
5	Build health systems readiness for NCDs	10 (8.4)	Sri Lanka,^37,38,42^ India,^32^ Bhutan,^29,30^ Bangladesh,^27,33,43^ Maldives^31^
6	Improve monitoring and accountability for quality of care	10 (8.4)	India,^32,35,45^ Nepal,^36^ Bangladesh,^33,43^ Afghanistan,^34^ Sri Lanka,^37^ Bhutan,^30^ Multi-country^7^
7	Strengthen human resources pool and distribution	9 (7.6)	India,^32,35,40^ Pakistan,^41^ Nepal,^36^ Bangladesh,^43^ Sri Lanka,^37^ Bhutan,^30^ Multi-country^47^
8	Enhance regulatory mechanisms for public-private partnership	9 (7.6)	Sri Lanka,^38^ Bhutan,^29,30^ Bangladesh,^26,33^ Afghanistan,^34^ India,^35,40^ Multi-country^47^
9	Enhance safety nets for the poor and vulnerable	9 (7.6)	Sri Lanka,^38^ Pakistan,^41^ Bhutan,^29^ Afghanistan,^25,34^ India,^40^ Maldives,^39^ Multi-country^8,47^
10	Invest in e-health	6 (5.0)	Nepal,^36^ India,^40^ Sri Lanka,^37^ Bhutan,^30^ Maldives,^31^ Multi-country^47^
11	Continue support from the international community	4 (3.4)	Nepal,^28^ Pakistan,^41^ Afghanistan^24,25^
12	Emphasize preventive care	4 (3.4)	Nepal,^36^ Bangladesh,^33^ Bhutan,^30^ Multi-country^47^
13	Improve UHC measurement and monitoring	3 (2.5)	Bhutan,^29^ Bangladesh,^44^ India^45^
14	Strengthen decentralized governance	3 (2.5)	Pakistan,^26,41^ India^40^

## Discussion

This scoping review presents a general mapping and key highlights of the existing literature on UHC challenges and opportunities in the South Asian region. As evident from the review, the number of publications examining UHC status and challenges has considerably increased during the past 10 years signaling heightened attention on UHC across countries in South Asia. However, there was a significant drop during the COVID-19 pandemic, highlighting the shift to emergent issues and the need to focus on immediate priorities at hand during the period. An extremely limited number of articles addressed UHC challenges comprehensively and along the lines of the globally agreed definition of UHC, though most articles addressed several independent components of UHC. This indicates a significant gap in the original research and highlights the need for comprehensive country-focused assessments of UHC. While quantitative assessments exist at a global level^9^ and there have been efforts with regional-level assessments,^7,8,13^ there is a need for a more nuanced evaluation of country progress, beyond progress measured in quantitative terms, to unravel stories behind UHC trends at country and regional levels.

Several new tools have emerged in the last few years^5,6^ that would both support country assessments and enable cross-country comparisons. This review uncovers significant disparities in methodological approaches and prioritization of UHC themes. Since UHC is a multi-dimensional concept and countries are expected to be in varying levels of the trajectory, the review attempts to summarize and highlight essential themes using WHO definition and guidance on UHC progress. This is expected to help inform similar assessments in the future and contribute to the discussion on the topic.

As anticipated, there are diverse and largely variable country-specific challenges to UHC that emerged in this review with several of them consistently present across countries in the region. Underfunding of the health system emerges as a critical challenge for the region often driven by larger issues beyond the health sector, such as political and economic stability along with the level of public sector engagement in health care. It corroborates that there is an increasing recognition of the political economy of UHC^48^ and the critical role of the public sector and public finance for UHC.^49,50^ While these issues may appear to sit largely beyond the health sector, the sector bears critical responsibilities in reemphasizing and advocating the linkage between better health and greater economic and social progress. Improvements in life expectancy and reduced disease burden would stimulate growth through lower fertility rates, higher investments in human capital, increased household savings, increased foreign investment, and greater social and macroeconomic stability.^51^ Similarly, domestic public funds are an essential component of a high-performing, sustainable, and equitable health financing system that can drive progress toward UHC^50,52^ and health sector needs to engage more meaningfully within the government to ensure that public financial management architecture and reforms are in sync with the goals and objectives of the health sector.^53^

Within the health systems, common challenges centered around fragmentation in the health system and services, weak regulatory oversight of the private sector, equality of access, shortages and maldistribution of health workers, suboptimal quality of care, and gaps in critical health interventions particularly related to NCDs. In terms of financial protection, the nascent stage or inadequate coverage of social protection or insurance systems was the more frequently reported challenge. In general, we observe that countries with a smaller and largely homogenous health system, such as Bhutan and Maldives reported more significant challenges related to service delivery bottlenecks, such as availability of resources or logistical difficulties in access. These countries continue to struggle to make adequate resources available, which are either not produced within their respective countries or faced with the delivery challenges posed by geographical and settlement patterns.^30,31^ In other countries such as Afghanistan, Bangladesh, India, Nepal, and Pakistan, besides the challenges in service delivery, issues with governance such as health system fragmentation, weak oversight of the private sector, and inability to institute a sound financial protection mechanism for the population emerge as critical priorities. Sri Lankan health system, which has been recognized internationally since the 1970s as a highly successful low-cost model, is faced with low public investments and resources for health, and the health system is ill-prepared to deal with the emerging challenge of NCDs.^37,42^

Reorganizing the service delivery architecture and developing a health system fit for purpose, with stronger stewardship of the government, fall squarely within the health sector leadership. Stewardship of the state is necessary for expanding access to health care in South Asia, particularly in view of making it more equitable.^54^ Other critical priorities are tackling the onslaught of NCDs and enhancing prioritization and investments in primary health care (PHC). As an essential foundation of UHC, robust PHC systems have profound potential to improve health outcomes while saving overall health care costs. Primary health care is efficient and equitable, contributes to building health security and resilience, and modest investments will be needed to reap the PHC dividend.^55^ With NCD-related deaths ranging from 44% to 84% out of the total number of deaths in South Asian countries,^56^ NCDs are a major priority in all countries of the region. Since PHC systems in most countries were traditionally designed for maternal and child health interventions and communicable diseases, both structural and systemic reforms are required to deliver screening programs, early detection, and provide comprehensive NCD care at the PHC level.^57^

These opportunities are supported by recommendations extracted from the papers compiled in this review. Among the prominent recommendations are service package and delivery redesign, increasing investment in health, revisiting health financing modalities, enhancing the role of public sector in health, and building health systems and readiness for NCDs. These recommendations are generally reflective of the adaptive and resilience approaches and mirror the broader recommendations for UHC and health system resilience in the literature.^10,55,49,50,52,53,57^ While acknowledging the limited generalizability of the recommendations, particularly considering the paramount consideration of country-specific contextual factors, they certainly provide an indication of the priority areas for the region. They can be considered as starting points for policy deliberations that can be further modified based on the countries’ political and macro-fiscal contexts, health systems architecture, health care needs, and demographics.

### Strengths and Limitations

This review maps a rapidly evolving literature on UHC challenges and opportunities in the South Asian region. To our knowledge, this is the first review to specifically examine UHC challenges and synthesize key policy messages for accelerating progress toward UHC in the region.

However, given the multidisciplinary and contextual nature of the topic of study, the review has several limitations. First, the dearth of studies examining UHC comprehensively led us to explore materials adopting a multiplicity of search designs, public reports, and perspectives wherein variability in quality of evidence in reviewed materials may have occurred. Second, some countries were completely absent in our systematic database search which reflects lapses in comparable methodological quality and availability of necessary information to allow identification or selection. We attempted to correct this by increasing the focus on the underrepresented countries in our manual and reference searches. Finally, we acknowledge that documentation of challenges and priorities may be more subtle than we anticipated and may also be happening in several non-traditional publication mediums, which we may have missed. In view of these limitations, our review should be seen as an initial attempt to bring together evidence and perspectives on the challenges and priorities for UHC in the South Asia region.

## Conclusion

A diversity of challenges, mostly driven by the country-specific context and health system architecture, continue to falter progress toward UHC in the South Asia region. While challenges are unique to specific countries, there are several consistent themes that emerge from this review: underfunding of the health system, fragmentation in the health system and the inability to effectively regulate the private sector, health system unprepared to effectively address NCDs, and a major concern on quality of and equality of access to health care. In addition, the nascent stage or inadequate coverage of social protection or insurance systems continues to drive a large number of individuals and households in countries in the region into financial hardship.

The paucity of country-driven literature on comprehensive assessments of UHC at national and subnational levels is attributed to inadequate data and tracking systems in most South Asian countries. Even where available, the methodological approaches are not consistent or standardized. For a region with a quarter of the world’s population, what happens in South Asia matters to the world. Efforts to reorganize service delivery systems emphasizing PHC, which incorporates the growing concern on NCDs, along with rigorous monitoring for quality of care and UHC accountability framework, are critical priorities. Commitment to increase investments and public sector engagement in the health sector supported by a sound health financing architecture holds significant promise to further this endeavor. Considering this as an initial attempt to bring together relevant evidence and perspectives on this issue, we recommend future research to investigate this further and examine how the challenges and priorities highlighted here evolve over time.

## Supplemental Material

sj-docx-1-aph-10.1177_10105395241296653 – Supplemental material for Challenges and Opportunities for Universal Health Coverage in South Asia: A Scoping Review Supplemental material, sj-docx-1-aph-10.1177_10105395241296653 for Challenges and Opportunities for Universal Health Coverage in South Asia: A Scoping Review by Jayendra Sharma, Milena Pavlova and Wim Groot in Asia Pacific Journal of Public Health
