# Supplementary material for: Challenges and Opportunities for Universal Health Coverage in South Asia: A Scoping Review
Source: Asia Pac J Public Health. 2024 Nov 15;37(1):7–16. doi: 10.1177/10105395241296653 (PMC11800725; doi:10.1177/10105395241296653)
Supplement: sj-docx-1-aph-10.1177_10105395241296653 – Supplemental material for Challenges and Opportunities for Universal Health Coverage in South Asia: A Scoping Review [file sj-docx-1-aph-10.1177_10105395241296653.docx]

**Supplementary file**

Contents:

1. Health and economic profile of South Asian countries, 2021 2
2. PRISMA-ScR Checklist 3
3. Search strings 6
4. Publications included in the scoping review 7
5. Scoping review protocol 15

**1. Health and economic profile of South Asian countries, 2021**

| Country | Population | UHC Service Coverage Index | Catastrophic incidence (10%)  2019 | GDP per capita (Current USD) | Life Expectancy at birth | Infant mortality rate | Physician density |
| --- | --- | --- | --- | --- | --- | --- | --- |
| Afghanistan | 40,099,464 | 41 | 26.1 | 1,674 | 62.0 | 43.4 | 0.3 |
| Bangladesh | 169,356,256 | 52 | 24.4 | 6,523 | 72.4 | 22.9 | 0.7 |
| Bhutan | 777,486 | 60 | 4.0 | 12,036 | 71.8 | 22.5 | 0.5 |
| India | 1,407,563,904 | 63 | 17.5 | 7,368 | 67.2 | 25.5 | 0.7 |
| Maldives | 521,457 | 61 | 10.3 | 20,707 | 79.9 | 5.1 | 2.1 |
| Nepal | 30,034,988 | 54 | 10.7 | 4,252 | 68.4 | 22.8 | 0.9 |
| Pakistan | 231,402,112 | 45 | 5.4 | 5,773 | 66.1 | 52.8 | 1.1 |
| Sri Lanka | 22,156,000 | 67 | 5.4 | 14,622 | 76.4 | 5.8 | 1.2 |

*Sources: World Bank World Development Indicators database 2023; UHC Global Monitoring Report 2023^9^*

**2. Preferred Reporting Items for Systematic reviews and Meta-Analyses extension for Scoping Reviews (PRISMA-ScR) Checklist**

| **SECTION** | **ITEM** | **PRISMA-ScR CHECKLIST ITEM** | **REPORTED ON PAGE #** |  |
| --- | --- | --- | --- | --- |
| **TITLE** | | | | |
| Title | 1 | Identify the report as a scoping review. | 1 |  |
| **ABSTRACT** | | | | |
| Structured summary | 2 | Provide a structured summary that includes (as applicable): background, objectives, eligibility criteria, sources of evidence, charting methods, results, and conclusions that relate to the review questions and objectives. | 1 |  |
| **INTRODUCTION** | | | | |
| Rationale | 3 | Describe the rationale for the review in the context of what is already known. Explain why the review questions/objectives lend themselves to a scoping review approach. | 2-3 |  |
| Objectives | 4 | Provide an explicit statement of the questions and objectives being addressed with reference to their key elements (e.g., population or participants, concepts, and context) or other relevant key elements used to conceptualize the review questions and/or objectives. | 3 |  |
| **METHODS** | | | | |
| Protocol and registration | 5 | Indicate whether a review protocol exists; state if and where it can be accessed (e.g., a Web address); and if available, provide registration information, including the registration number. | Supplementary file, p 15 |  |
| Eligibility criteria | 6 | Specify characteristics of the sources of evidence used as eligibility criteria (e.g., years considered, language, and publication status), and provide a rationale. | 4-5 |  |
| Information sources* | 7 | Describe all information sources in the search (e.g., databases with dates of coverage and contact with authors to identify additional sources), as well as the date the most recent search was executed. | 4 |  |
| Search | 8 | Present the full electronic search strategy for at least 1 database, including any limits used, such that it could be repeated. | Supplementary file, p 6 |  |
| Selection of sources of evidence† | 9 | State the process for selecting sources of evidence (i.e., screening and eligibility) included in the scoping review. | 3-4 |  |
| Data charting process‡ | 10 | Describe the methods of charting data from the included sources of evidence (e.g., calibrated forms or forms that have been tested by the team before their use, and whether data charting was done independently or in duplicate) and any processes for obtaining and confirming data from investigators. | 4-5 |  |
| Data items | 11 | List and define all variables for which data were sought and any assumptions and simplifications made. | 4-5 |  |
| Critical appraisal of individual sources of evidence§ | 12 | If done, provide a rationale for conducting a critical appraisal of included sources of evidence; describe the methods used and how this information was used in any data synthesis (if appropriate). | NA |  |
| Synthesis of results | 13 | Describe the methods of handling and summarizing the data that were charted. | 5 |  |
| **RESULTS** | | | | |
| Selection of sources of evidence | 14 | Give numbers of sources of evidence screened, assessed for eligibility, and included in the review, with reasons for exclusions at each stage, ideally using a flow diagram. | 5 |  |
| Characteristics of sources of evidence | 15 | For each source of evidence, present characteristics for which data were charted and provide the citations. | 5, Supplementary file, p 7 |  |
| Critical appraisal within sources of evidence | 16 | If done, present data on critical appraisal of included sources of evidence (see item 12). | NA |  |
| Results of individual sources of evidence | 17 | For each included source of evidence, present the relevant data that were charted that relate to the review questions and objectives. | Supplementary file, p 7 |  |
| Synthesis of results | 18 | Summarize and/or present the charting results as they relate to the review questions and objectives. | 6-8 |  |
| **DISCUSSION** | | | | |
| Summary of evidence | 19 | Summarize the main results (including an overview of concepts, themes, and types of evidence available), link to the review questions and objectives, and consider the relevance to key groups. | 11 |  |
| Limitations | 20 | Discuss the limitations of the scoping review process. | 10-11 |  |
| Conclusions | 21 | Provide a general interpretation of the results with respect to the review questions and objectives, as well as potential implications and/or next steps. | 11 |  |
| **FUNDING** | | | | |
| Funding | 22 | Describe sources of funding for the included sources of evidence, as well as sources of funding for the scoping review. Describe the role of the funders of the scoping review. | NA |  |

**3. Search strings**

**Pubmed**

("Universal Health Insurance"[MeSH Terms] OR ("universal health coverage"[Title/Abstract] OR "uhc"[Title/Abstract] OR "universal health care"[Title/Abstract] OR "universal healthcare"[Title/Abstract] OR "health coverage"[Title/Abstract] OR "universal health insurance"[Title/Abstract] OR "universal coverage"[Title/Abstract])) AND ("south asia"[Title/Abstract] OR "southasia"[Title/Abstract] OR "south asia"[Title/Abstract] OR " Asia, Southern"[MeSH Terms] OR "Afghanistan"[Title/Abstract] OR "Bangladesh"[Title/Abstract] OR "Bhutan"[Title/Abstract] OR "India"[Title/Abstract] OR "Maldives"[Title/Abstract] OR "Nepal"[Title/Abstract] OR "Pakistan"[Title/Abstract] OR "Sri Lanka"[Title/Abstract] OR "Afghanistan"[MeSH Terms] OR "Bangladesh"[MeSH Terms] OR "Bhutan"[MeSH Terms] OR "India"[MeSH Terms] OR "Maldives"[MeSH Terms] OR "Nepal"[MeSH Terms] OR "Pakistan"[MeSH Terms] OR "Sri Lanka"[MeSH Terms])

**Scopus**

( TITLE-ABS-KEY ( "universal health coverage"  OR  uhc  OR  "universal health care"  OR  "universal healthcare"  OR  "health coverage"  OR  "universal health insurance"  OR  "universal coverage" )  AND  TITLE-ABS-KEY ( "South Asia"  OR  southasia  OR  south-asia  OR  afghanistan  OR  bangladesh  OR  Bhutan

OR  india  OR  maldives  OR  nepal  OR  pakistan  OR  "Sri Lanka" ) )

**Web of Science**

(TS=("universal health coverage" OR uhc OR "universal health care" OR "universal healthcare" OR "health coverage" OR "universal health insurance" OR "universal coverage")) AND TS=("South Asia" OR southasia OR south-asia OR Afghanistan OR Bangladesh OR Bhutan OR India OR Maldives OR Nepal OR Pakistan OR "Sri Lanka")

**4. Publications included in the scoping review**

| **Citation Ref #** | **Author, year** | **Country** | **Type of publication** | **Aim and objective** | **Key conclusions/authors' recommendations** |
| --- | --- | --- | --- | --- | --- |
|  |  |  |  |  |  |
| 46 | Ali & Rais, 2021 | Pakistan | Perspective | Investigate elite power struggles to explain ad hoc policymaking, instability, patronage politics, and rent-seeking in distribution of health care resources | - Political consolidation in health policy - Context based health policy and service delivery system with government in control - Need for better consultation and stakeholder involvement in health policies |
| 42 | Chapman and Dharmaratne, 2019 | Sri Lanka | Review | Highlight the challenges of achieving and maintaining UHC | - Devote more attention to NCDs - Enhance investments in health - Enhance the role of public sector in health |
| 38 | de Silva et al., 2016 | Sri Lanka | Perspective | Assess progress towards, and potential threats to, attaining UHC and the SDG for health in Sri Lanka | - Set higher and more ambitious targets - Inter-ministerial/inter-sectoral coordination of activities - Strengthen NCD services focussing on prevention and targeting working age men - Rehabilitative domiciliary care and hospices for palliative care Manage public-private mix in financing with appropriate safety nets |
| 44 | Gupta and Shahabuddin, 2018 | Bangladesh | Review | Compare Bangladesh’s UHC monitoring framework with the global-level recommendations | - Provide an explicitly defined framework with a comprehensive set of indicators - Include indicators related to palliative care, mental health, cataract surgery, neglected tropical diseases, and measurement of service need |
| 34 | Higgins-Steele et al., 2018 | Afghanistan | Viewpoint |  | - Primary health care must be the focus of UHC: improve quality of governance, stronger public sector administration and provider accountability, committing to UHC, improving the quality of services and ensuring a fairer distribution of access to care and health outcomes - Health financing reform: Introduce/expand risk pooling, increasing public financing for health, decreasing OOP, and further development of its purchasing and stewardship functions |
| 27 | Joarder et al., 2019 | Bangladesh | Qualitative study | Review the health policy environment and challenges for UHC | - Redesigning the public financial management structure - Introducing health-financing reform - Improving regulatory and mediatory mechanisms - Embracing intersectoral collaboration - Garnering political commitment for UHC |
| 36 | Pokharel and Silwal, 2018 | Nepal | Review | Analyze the design, expected benefits and challenges of realizing the goals of UHC through the recently launched SHI | - Expedite implementation of SHI - Efficient health care delivery system, adequate human resources for health, a strong information system, improved transparency and accountability, and a balanced mix of the preventive, promotive, curative, and rehabilitative services including actions to address the social determinants of health. - Institute facility‐based accreditation system for quality assurance - Enhance transparency and accountability in the health sector |
| 45 | Prinja et al., 2017 | India | Research | To develop a metric for measuring the extent of UHC | - Careful inclusion of other indicators of service coverage - More work needs to be done to incorporate quality in the measurement framework. - Measurement of UHC should be done using both service coverage and financial risk protection |
| 7 | Rahman et al., 2017 | Afghanistan. Bangladesh, India, Nepal and Pakistan | Research Population-based multi-country | Investigate progress toward UHC in 5 South Asian countries | - Strong political commitment - Increased government spending on health - Improved service delivery - Proper monitoring of subsidized programs, ensuring standardized costs for both official and unofficial fees across all public facilities - Reconsidering both the demand side (committing to proper risk-pooling mechanisms for the whole population, expanding benefits, and reducing cost-sharing) and the supply side (expansion of infrastructure, human resources for health, and health services) |
| 43 | Rahman et al., 2018 | Bangladesh | Research | Investigate trends in the coverage of health services and financial risk protection in Bangladesh | - Increase spending on health at a minimum rate of 0·3% of GDP per year - Prioritize health in budget allocations - Incorporation of risk pooling health financing mechanisms - Prioritize prevention programmes for NCDs by strengthening the capacity of human resources and health facilities - Adequate supplies of drugs for NCDs, increased human resources for health, improved quality of care, and the establishment of more community clinics and other public health facilities in rural areas |
| 28 | Ranabhat et al., 2019 | Nepal | Review | Explore the challenges and opportunities for UHC in Nepal | - Strong government stewardship - Support of stakeholders and fair contribution - Distribution of resources by appropriate health financing modality |
| 8 | Scammell et al., 2016 | Afghanistan. Bangladesh, Bhutan, India, Maldives, Nepal, Pakistan and Sri Lanka | Review | Review of UHC for mothers and children in the 8 countries of South Asia | - Removing financial and non-financial barriers to accessing and receiving high-quality healthcare - Increasing the amount of investment in essential health services, while ensuring effective and efficient spending - Prioritising equity by allocating new resources to benefit the poorest first. |
| 41 | Shaikh and Ali, 2023 | Pakistan | Perspective/debate |  | - Increase budgetary allocation for health - Build health system readiness through strengthening human resources and availability of essential services - Establish safety nets for health detached from political interests - Decrease the reliance on donors’ funding - Enhance accountability - Strengthen and empower the district health system - Periodically review the UHC benefits package |
| 29 | Sharma et al., 2014 | Bhutan | Mixed methods case study | Assessment of UHC in Bhutan | - Match health spending to the growth of the national economy - Continued, perioding monitoring of financial protection - Strengthen system capacity to respond to the rise in NCDs - Clarify health policy or regulatory response for engagement of private sector - Clarify standards/packages of services - Address health equity - Subnational tracking of the UHC index is necessary |
| 32 | Sharma and Popli, 2023 | India | Review | Review of UHC in India | - Need to rapidly ramp up the infrastructure and skilled workforce  A strong political will towards UHC - An integrated approach to overcoming the inherent structural challenges of the system - Strengthen screening, preventative care, and long-term management of NCDs |
| 13 | Wang et al., 2018 | Bangladesh, Bhutan, India, Maldives, Nepal, Sri Lanka, Thailand & Timor-Leste | Quantitative | Assessment of financial protection status in 8 countries | - More effective health policies are needed to provide better financial protection of households |
| 40 | Kalita et al., 2023 | India | Qualitative study | Understand views of policy actors on conceptualizations of UHC, main barriers to realizing UHC, and policy strategies to address these barriers. | - More government budgetary allocation to health and mixed financing mechanisms - Prepayment and risk pooling to eliminate, or at least reduce, OOPE at the point of care and CHE - Expanding the benefits package of national insurance to include outpatient services - Adopt active and strategic purchasing - Consider both the public and private sectors in designing reforms - Planning HRH to a more nuanced and strategic approach - Reorganizing the health delivery system through gatekeeping and enhanced referral networks - Digital technologies to deliver better quality care and address gaps in access - Strengthen decentralized decision-making, program design, and implementation of health policies |
| 26 | Khan et al., 2023 | Pakistan | Editorial |  | - Increase healthcare funding - Equitable allocation of resources - Strengthen the implementation of Sehat Sahulat Program (UHC initiative) |
| 37 | Rajapaksa et al., 2021 | Sri Lanka | Report | Country-based reports that provide a detailed description of a health system and of policy initiatives in progress or development. | - Prioritize NCDs - Strengthen planning and projections to anticipate and respond to emerging health needs - Implement a comprehensive electronic health information system - Adopt targeted and innovative approaches for mother and child health - Increase government spending on the health sector - Transformative educational approaches for health workers - More effective stewardship role by the Ministry of Health - Address social and economic determinants of health |
| 33 | Ahmed et al., 2015 | Bangladesh | Report | Country-based reports that provide a detailed description of a health system and of policy initiatives | - Need for a realistic plan for an affordable payment mechanism for health in order to reduce catastrophic out-of-pocket payments for health and to develop parallel strategies for investing more in health. - Establish linkages between urban health services and the health ministry, referral systems, deployment of adequately-qualified providers - Increasing focus on primary health care and prevention strategies for both acute and long-term care - Evaluate some of the current vertical programmes for health systems strengthening - Prioritize investments in NCDs - Bring informal sector providers under the mainstream health systems and monitor their services |
| 35 | Selvaraj et al., 2022 | India | Country-based peer-reviewed reports based on a standardized template | Country-based reports that provide a detailed description of a health system and of policy initiatives | - Increase public investments (double in five years) - Integration of existing insurance systems - Professional councils to be overhauled for improved medical education and practice - Creation of parallel public health cadre - Emphasis on quality and accountability - Stronger regulations for controlling costs and quality in the private sector |
| 30 | Thinley et al., 2017 | Bhutan | Report | Country-based reports that provide a detailed description of a health system and of policy initiatives | - Examine public investments for health and explore alternative sources of finance - Assess private sector engagement in health - Strengthen the tertiary care level and bring in a good balance of primary, secondary and tertiary care levels - Invest more on primary prevention and interventions - Develop a comprehensive, robust and integrated system to monitor health-care quality and patient safety - Establish gatekeeping mechanism to promote efficient use of resources by different levels of health facilities. - Revitalize telemedicine - Establish, strengthen and sustain institutional capacities to conduct health technology assessment - Promote use of data for decision making - Explore mechanisms to retain health workers - Promote multisectoral action, particularly to combat the rising threat of NCDs |
| 25 | Ministry of Public Health, 2019 | Afghanistan | Government Report |  | Strategic directions:   - Mobilize domestic and external financing for health - Provide financial protection against catastrophic health expenditure and avoid impoverishment of families caused by OOP spending - Improve purchasing mechanisms - Improve efficiency and equity of public spending - Strengthen governance for health financing |
| 39 | Moosa and Usman 2020 | Maldives | Review | Explore how the national health expenditure is directed towards achieving UHC | - Prioritising spending to critical health issues such as maternal and child health, NCDs and infection control - Investments in empowering women and social support mechanism - Health financing policy needs to find a balance between the political motives and the health needs of the population |
| 31 | Eya et al., 2023 | Maldives | Commentary |  | - Telemedicine. - Strengthen health workforce. - Upgrade hospitals in different regions of the country.   Consider the threats of climate change. |
| 24 | Safi et al., 2022 | Afghanistan | Comment |  | - Long term: increased domestic resources to health services. - Short term: co-financing mechanisms and other strategies - Continuous support from the international community for provision of health services |
| 47 | Qarani and Kanji, 2015 | Afghanistan Pakistan | Review | Analyze the health system of two neighboring countries; Pakistan and Afghanistan | - Strategies to retain human resource within the country - Strengthen linkages between vertical and horizontal programs Introduce e-health - Strong monitoring system and quality assurance to reduce corruption, enhance transparency - Increase public sector spending on health - Emphasize on preventive measures - Public-private partnership - Programme aggressive safety nets |

**5. Scoping review protocol**

**Introduction**

The South Asia region, defined by the membership of the South Asian Association for Regional Cooperation (SAARC), comprises eight countries – Afghanistan, Bangladesh, Bhutan, India, Maldives, Nepal, Pakistan and Sri Lanka. The region is home to about a quarter of the world’s population. Despite global momentum and commitments on Universal Health Coverage, South-Asian countries continue to register low levels of coverage of essential health services and financial risk protection, while inequality in access to health care remains a serious issue for most countries. Without significant progress in these countries, global progress on UHC and Sustainable Development Goals would be severely challenged.

**Research objectives**

The objectives of this scoping review are to:

1. Examine the challenges and opportunities for UHC in South Asia
2. Identify key policy options for UHC in South Asia

**Methods**

We plan to use Arksey and O’Malley’s six-stage methodological framework for scoping reviews^1^. This method sequences the review into five stages, described below:

1. *Identifying the research question*

We will adopt the Population, Concept and Context (PCC) model, which is recommended as a guide to identify the main concepts in the research question, clarify inclusion criteria and inform the search strategy.^2^

| Population (P) | All residents of all ages |
| --- | --- |
| Context (C) | National level: single country, multiple countries, or collectively, the South Asia region |
| Concept (C) | Definition of UHC by the WHO; Country-specific definition of UHC where they exist |

1. *Identifying relevant studies*

*Inclusion/Exclusion:*

|  | Inclusion | Exclusion |
| --- | --- | --- |
| Article type | Peer-reviewed journal articles, studies and reports that are globally standardized by World Health Organization or a policy document from a national government are included  All study designs are considered. |  |
| Scope | Articles and reports needed to address the overall UHC progress and challenges or at least one of the identified thematic components. | Articles that focused on a specific aspect of UHC, examined a single or a set of specific disease or health system issue  Articles focused on sub-national levels |
| Year |  | Studies published prior to 2010 |

*Search strategy:*

The search strategy will follow multiple steps of development and peer review by a Librarian. The initial step has been undertaken and involved a preliminary search of one online database (Pubmed) below. This search resulted in 676 studies. In addition, we will examine webpages of ministries of health and multilateral institutions and Google search for any additional country-specific publications.

("universal health coverage"[Title/Abstract] OR uhc[Title/Abstract] OR "universal coverage"[Title/Abstract]OR "universal health"[Title/Abstract]) AND ("South asia"[Title/Abstract] OR southasia[Title/Abstract] OR south-asia[Title/Abstract] OR Afghanistan[Title/Abstract] OR Bangladesh[Title/Abstract] OR Bhutan[Title/Abstract] OR India[Title/Abstract] OR Maldives[Title/Abstract] OR Nepal[Title/Abstract] OR Pakistan[Title/Abstract] OR "Sri Lanka"[Title/Abstract])

1. Study selection

Studies retrieved through the searches will be imported into EndNote 20 for subsequent cleaning and management. We will apply three stages screening screening process for the selection of studies. Stage 1 will involve removal of duplicates, non-relevant publications, and publications that were outside the stipulated time frame. Stage 2 screening involvs reading titles and abstracts of all publications that passed Stage 1 in order to assess their relevance to the topic based on the inclusion criteria. In Stage 3, the full texts of the publications that passed Stage 2 screening will be assessed against the inclusion criteria for the final selection of studies to be included in the review. We plan to engage independent reviewers at every stage of the process.

1. Charting the data

Data will be sorted and charted by author(s), publication year, country names. In addition, the publications will be examined and classified according to the pre-defined themes that emerge from the definitions adopted. We have pre-identified the following themes through a limited literature review: (1) health policy and governance; (2) effective coverage of services; (3) financial protection; (4) equity of access; and, (5) UHC data and monitoring. A standardized data extraction tool will be developed in Microsoft Excel and pilot-tested to chart the results.

1. Collating, summarizing and reporting the results.

Our report structure and reporting of the results will be guided by the Preferred Reporting Items for Systematic reviews and Meta-analysis extension for Scoping Reviews (PRISMA-SCR) checklist^3^ to structure and report the review results. Simple descriptive analysis will be performed to profile the articles summarizing the origin, time, objective and design of the studies or reports. We will then delve into basic qualitative content analysis of the findings along the identified themes/categories.^4^

**Conclusion**

Progress towards UHC would entail deeper understanding of the contextual challenges to identify relevant policy opportunities. South Asia has been struggling on the UHC trajectory and there is a need to understand the available evidence and identify the contextual challenges and approaches to inform policy. This review aims to generate evidence to guide research and inform policies. The results could assist governments, development partners, academia and other stakeholders to identify priority areas for UHC strategies in South Asia.

**Ethics and dissemination**

We do not foresee the requirement for ethical clearance of this study. Our dissemination strategy includes peer review publication, presentation at conferences and to relevant stakeholders.

**References:**

1. Arksey H, O’Malley L. Scoping studies: towards a methodological framework. Int J Soc Res Methodol. 2005;8(1):19–32.

2. Peters MDJ, Marnie C, Tricco AC, et al. Updated methodological guidance for the conduct of scoping reviews. JBI Evid Synth. 2020 Oct;18(10):2119-2126.

3. Tricco AC, Lillie E, Zarin W, et al. PRISMA Extension for Scoping Reviews (PRISMA-ScR): Checklist and Explanation. Ann Intern Med. 2018; 169(7):467–73.

4. Pollock D, Peters MDJ, Khalil H, et al. Recommendations for the extraction, analysis, and presentation of results in scoping reviews. JBI Evid Synth. 2023 Mar 1;21(3):520-532.
